# Strategies for becoming a more desirable mate: evidence from Lithuania

**DOI:** 10.3389/fpsyg.2024.1437393

**Published:** 2024-09-30

**Authors:** Menelaos Apostolou, Mark Sullman, Rasa Markšaitytė, Laura Šeibokaitė, Timo Juhani Lajunen

**Affiliations:** ^1^Department of Social Sciences, University of Nicosia, Nicosia, Cyprus; ^2^Department of Psychology, Vytautas Magnus University, Kaunas, Lithuania; ^3^Department of Psychology, Norwegean University of Science and Technology, Trondheim, Norway

**Keywords:** mating strategies, attraction, mating, mate choice, sex differences

## Abstract

**Introduction:**

Mate-seekers employ several strategies to become more attractive as mates. In the present study, we examined the use of 10 strategies for becoming more desirable as a mate in the Lithuanian cultural context.

**Methods:**

Using a sample of 295 Lithuanian-speaking participants, we explored the frequency and types of strategies employed to enhance mate appeal.

**Results:**

The most frequently used strategy was “Enhance looks,” followed by “Show off abilities and talents” and “Demonstrate similarity.” The least used strategies were “Show off and exaggerate wealth” and “Drastic appearance changes.” The 10 strategies could be classified into two domains or main strategies, with participants indicating a more frequent use of “Develop and demonstrate desirable traits” followed by “Deceive about undesirable traits.” Additionally, sex differences and age differences were identified for several strategies.

**Discussion:**

The findings highlight the prominence of certain strategies over others in the Lithuanian context, with a notable emphasis on developing and demonstrating desirable traits. The study also reveals variations in strategy use based on sex and age, suggesting that these factors influence mate-seeking behaviors.

## Introduction

People do not choose mates randomly; they have well-defined preferences that guide their choices (Buss and Schmitt, 2019). For instance, they tend to prefer mates who are good-looking, enjoy good health, and have a high social status (Buss, 2016). Thus, when they look for mates, individuals adopt specific strategies which aim to make themselves more desirable as mates by addressing these preferences (Buss, 1988; Schmitt and Buss, 1996). The purpose of the current study is to examine these strategies in the Lithuanian cultural context.

### Strategies for appealing as a prospective mate

Intimate relationships enhance survival and reproductive success by providing support and increasing the likelihood of raising offspring, which are strong evolutionary drivers for selective mate choice (Buss, 2016; Fisher, 2017). We need to say however that this argument may not fully apply to Western societies, where many relationships do not lead to reproduction and where not all relationships are heterosexual [see also Bártová and Štěrbová (2020)]. Mate-seekers prioritize partners who can significantly contribute to their fitness, favoring those with traits that suggest good resource provision, such as wealth, intelligence, and social status (Buss and Barnes, 1986; Buss et al., 2001; Thomas et al., 2020; Walter et al., 2020). Additionally, traits indicating reproductive potential, like youth and health, are highly valued, often reflected in physical attributes like smooth skin and symmetry (Li et al., 2011; Thomas et al., 2020; Walter et al., 2020). Personality traits such as kindness, reliability, and emotional stability are also crucial (Buss et al., 2001; Walter et al., 2020). Furthermore, there is a preference for similarity, with individuals seeking partners who share similar desirable traits (Apostolou and Polycarpou, 2023; Luo, 2017). It is important to note at this point that preferences are not always fully conscious (Miller and Todd, 1998; Wood and Brumbaugh, 2009), and there is often a considerable discrepancy between stated preferences and actual mate selection (Kučerová et al., 2018). Overall, the selection of a mate is influenced by preferences for physical attractiveness, resource availability, personality, and similarity.

Human mating involves strategic behaviors aimed at enhancing mating success by aligning with prospective partner’s preferences (Apostolou et al., 2021; Gangestad and Simpson, 2000). Specifically, in light of what people prefer in a mate, we expect that individuals would employ strategies that enhance their looks, demonstrate their capacity to control or generate resources, demonstrate their good character qualities, and demonstrate similarity with their partners. For instance, mate-seekers may buy an expensive car to demonstrate their wealth. People may also use strategies that aim to exaggerate or even fabricate qualities to appear more desirable than they are, such as using plastic surgery to look younger or overstating personal achievements (Buss, 2016; Schmitt and Buss, 1996).

Moreover, there are specific sex differences in mate preferences. In particular, women generally prioritize a partner’s ability to acquire resources, while men often value physical attractiveness more (Buss et al., 2001; Li et al., 2011; Thomas et al., 2020; Walter et al., 2020). Note however, that the magnitude of the observed sex differences is relatively small for instance, men and women do not differ very much in the value they ascribe to the good looks of a prospective mate (Walter et al., 2020). These sex differences suggest that there would also be sex differences in the strategies people adopt to become more desirable as mates. Specifically, women would be more likely to employ strategies enhancing their appearance, whereas men would be more likely to focus on demonstrating their resource acquisition capabilities. Current literature provides support for these arguments.

### Current literature

Across different cultures, individuals enhance their physical appearance through weight loss, exercise, and muscle building (Kowal et al., 2022; McCabe et al., 2009; Shomaker and Furman, 2010). Women often use makeup and cosmetics to improve facial appearance (Mafra et al., 2020), while men focus on increasing muscle mass (Gosse and Arnocky, 2012; Vartanian et al., 2012). Both sexes engage in cosmetic surgeries to boost their attractiveness, with women typically investing more time and resources in these enhancements (Arnocky, 2016).

Research has also explored broader strategies for becoming more appealing as mates. Buss (1988) initially identified 101 behaviors grouped into 23 major strategies for attracting mates, which Schmitt and Buss (1996) later expanded to 130 behaviors and 31 strategies. These strategies include demonstrating good character through actions like honesty and kindness, enhancing physical attractiveness, and showcasing resource provision capabilities (Bendixen and Kennair, 2015). More recently, Apostolou et al. (2021) conducted a study with 326 Greek-speaking participants using qualitative methods to identify 87 mate-attracting acts. A larger sample of 2,197 participants enabled categorizing these into 16 strategies through exploratory factor analysis. Key strategies included enhancing appearance, showing off abilities and wealth, and developing similar interests. Notably, no strategies explicitly aimed at demonstrating good character emerged. The study also found sex differences, with men more likely to display resource acquisition strategies and women more focused on improving their looks.

A subsequent study employed the instrument developed by Apostolou et al. (2021) to examine the strategies that people use to become more appealing as mates in a large cross-cultural sample (Apostolou et al., 2024). Specifically, using a sample of 7,181 participants from 14 different countries, the study identified 10 different strategies. The most frequently used strategy was “Enhance looks,” followed by “Show off abilities and talents” and “Demonstrate similarity.” On the other hand, the least frequently used strategies were “Keep undesirable things hidden,” “Show off and exaggerate wealth and abilities,” and “Drastic appearance changes.” Sex differences were also found. In particular, female participants indicated more extensive use of the “Enhance looks” strategy than male participants, while male participants indicated a more extensive use of the “Increase income and social status” and “Show off and exaggerate wealth and abilities” strategies than female participants. In turn, by using second-order factor analysis, the 10 strategies were classified into two main strategies, namely “Develop and demonstrate desirable traits” and “Deceive about undesirable traits.” Although there were differences in the mean scores, the factor structure of the identified strategies was generally consistent across different cultures.

### The current study

The existing literature offers good insight into the strategies that people use to become more appealing as mates. However, additional research in the area is required to examine the consistency of these strategies across different cultural settings. Accordingly, the present study aimed to contribute to the existing literature by examining the strategies that people use to become more appealing as mates identified by Apostolou et al. (2024) in the Lithuanian cultural context. More specifically we predict:

*H1:* In the Lithuanian Context, Strategies for Enhancing Mate Appeal Align with the 10-Factor, Two-Domain Structure Identified by Apostolou et al. (2024).

*H2:* Women would be more likely to employ strategies enhancing their appearance, whereas men would be more likely to focus on demonstrating their resource acquisition capabilities.

## Methods

### Participants

The study took place at a public university located in Lithuania and received clearance from the institution’s ethics review board. Participants were recruited by forwarding the study link to students and colleagues and by advertising it on social media, including Facebook and Instagram. A pilot study indicated that the questionnaire took about 10 min to complete. Participants did not receive any reimbursement for participating. We excluded the responses of six participants who completed less than 80% of the questionnaire. In total, 295 Lithuanian-speaking individuals (236 women, 58 men, and one participant who did not indicate their sex) took part. The mean age of women was 31.3 years (*SD* = 11.2, Min = 18, Max = 69, Range = 51), and the mean age of men was 30.5 years (*SD* = 12.4, Min = 18, Max = 72, Range = 54). Moreover, 39.3% of the participants were in a relationship, 31.9% were married, 24.1% were single, and 4.7% indicated their relationship status as “other.”

### Materials

The original instrument developed by Apostolou et al. (2021) was translated into Lithuanian through the back-translation method. The survey was created using Google Forms and conducted online. It consisted of two parts. In the first part, participants were asked the following: “Please indicate to what extent you have done each of the following in the past to become more desirable as a mate.” Subsequently, they were given 87 acts such as “I was showing off my abilities and skills,” and “I was wearing clothes that flattered me” identified by Apostolou et al. (2021) to rate on the following scale: 1 - Not at all, 5 - Very extensively. In the second part, demographic information was collected, including sex, age, and relationship status (single, in a relationship, married, other).

### Data analysis

Apostolou et al. (2024) employed the instrument developed by Apostolou et al. (2021) to investigate strategies for becoming more desirable as a mate in a sample drawn from 14 nations. Using exploratory factor analysis on the pooled sample, they classified the 87 acts of the instrument into 10 strategies and two main strategies. Subsequently, through confirmatory factor analysis, they found that the identified factor structure was generally consistent across the different nations in the sample. In the current study, we aimed to examine whether the factor structure identified by Apostolou et al. (2024) would fit the Lithuanian sample. For this purpose, we performed confirmatory factor analysis using the JASP 0.18.3 software.

As our variables were measured using a Likert scale, we employed the Diagonally Weighted Least Squares (DWLS) estimation method (Li, 2016). To assess goodness of fit, we employed the following indexes: The Comparative Fit Index (CFI) is considered very good if it is equal to or greater than 0.95, good if it falls between 0.90 and 0.95, acceptable if it is between 0.80 and 0.90, and poor if it is less than 0.80; the Standardized Root Mean Square Residual (SRMR) - acceptable range being between 0 and 0.08; the Root Mean Square Error of Approximation (RMSEA) - values less than 0.05 are good, values between 0.05 and 0.08 are acceptable, values between 0.08 and 0.1 are marginal, and values greater than 0.1 are poor. Furthermore, to identify significant effects, we performed a series of MANCOVA tests on each strategy using SPSS version 28. More specifically, we employed Apostolou et al.’s (2024) classification, dividing the 87 acts into 10 strategies. Thus in order to examine the effects of age and sex, we performed a MANCOVA test, placing the different acts that composed each strategy as the dependent variables, and sex as the categorical and age as the continuous independent variable. The analysis was repeated for each strategy, so in total 10 MANCOVA tests were performed.

## Results

### Factor structure

Confirmatory factor analysis indicated that the 10-strategy structure identified by Apostolou et al. (2024) was also consistent for the current sample. More specifically, the CFI was 0.978 and the RMSEA was 0.003. However, these two indices may not give accurate fit information when the DWLS estimation method is used, with the SRMR being a more robust index (Shi and Maydeu-Olivares, 2020). The SRMR was 0.075, indicating a good fit. We repeated the process for the second-order structure (two main strategies). Here, the CFI indicated a moderate fit (0.858), the RMSEA a poor fit (0.125), and the SRMR a good fit (0.063).

### Mean scores

To identify which strategy participants were more likely to use, we calculated the means and the standard deviations for each of the 10 strategies, and placed them in a hierarchical order in Table 1. The most frequently used strategy was “Enhance looks,” followed by “Show off abilities and talents” and “Demonstrate similarity.” In the middle of the hierarchy were “Do more physical exercise and sports,” “Lose weight,” “Increase income and social status,” and “Keep undesirable things hidden.” At the bottom of the hierarchy were “Enhance social media profile,” “Show off and exaggerate wealth,” and “Drastic appearance changes.” With respect to the two main strategies, “Develop and demonstrate desirable traits” was much more frequently used (*M* = 2.83, *SD* = 0.72) than “Deceive about undesirable traits” (*M* = 1.88, *SD* = 0.66).

**Table 1:** Mean scores, sex, and age effects.

Strategies	Overall	Women	Men	Sex			Age		
	Mean (SD)	Mean (SD)	Mean (SD)	Pillai’s trace	*p-*value	η_p_*^2^*	Pillai’s trace	*p-*value	η_p_*^2^*
Enhance looks	3.66 (0.81)	3.81 (0.74)	3.09 (0.83)	0.250	<0.001	0.250	0.097	(−) 0.002	0.097
Show off abilities and talents	3.22 (0.92)	3.29 (0.90)	2.98 (0.98)	0.030	0.187	0.030	0.088	(−) <0.001	0.088
Demonstrate similarity	3.00 (0.90)	3.00 (0.89)	3.00 (0.95)	0.129	<0.001	0.129	0.209	(−) <0.001	0.201
Do more physical exercise and sports	2.63 (1.19)	2.58 (1.18)	2.86 (1.20)	0.010	0.391	0.010	0.016	0.191	0.016
Lose weight	2.53 (1.25)	2.64 (1.28)	2.08 (1.06)	0.225	<0.001	0.225	0.004	0.779	0.004
Increase income and social status	2.50 (1.01)	2.45 (1.00)	2.75 (1.02)	0.052	0.031	0.052	0.048	(+) 0.048	0.048
Keep undesirable things hidden	2.49 (1.06)	2.56 (1.09)	2.26 (0.92)	0.049	0.006	0.049	0.060	(−) 0.001	0.060
Enhance social media profile	2.26 (1.30)	2.42 (1.32)	1.62 (0.93)	0.098	<0.001	0.098	0.082	(−) <0.001	0.082
Show off and exaggerate wealth and abilities	1.76 (0.87)	1.75 (0.87)	1.84 (0.92)	0.046	0.060	0.046	0.046	0.058	0.046
Drastic appearance changes	1.38 (0.45)	1.39 (0.41)	1.36 (0.58)	0.102	0.007	0.102	0.097	(−) 0.010	0.097

The signs in parenthesis indicate the direction of the relationship. A negative sign indicates that older participants gave lower scores than younger participants, while a positive sign indicates that they gave higher scores.

### Significant effects of sex and age

In our analysis, we performed 10 different MANCOVA tests, and to avoid increasing the probability of committing a Type I error, we performed Bonferroni corrections, setting the alpha level to 0.005 (0.050/10). As we can see from Table 1, a significant main effect of sex was found for several strategies. As indicated by the effect size, the largest effect was for “Enhance looks,” followed by “Lose weight,” with women giving higher scores than men. In both cases, the effect sizes were considerable, indicating a large difference in the use of these strategies between men and women. A moderate to large sex difference was found for “Enhance social media profile,” with women also giving higher scores than men. For most of the strategies, there was a significant main effect of age. As indicated by the effect size, the largest effect was for “Demonstrate similarity,” followed by “Enhance looks” and “Show off abilities and talents.” In all cases, the regression coefficient was negative, indicating that older participants reported using these strategies less than younger ones.

## Discussion

In the current study, we examined the use of 10 strategies for becoming more desirable as a mate in the Lithuanian cultural context. We found that the most frequently used strategy was “Enhance looks,” followed by “Show off abilities and talents” and “Demonstrate similarity.” The least used strategies were “Show off and exaggerate wealth” and “Drastic appearance changes.” The 10 strategies could be classified into two domains or main strategies, with participants indicating a more frequent use of “Develop and demonstrate desirable traits” followed by “Deceive about undesirable traits.” Sex differences and age differences were identified for several strategies.

Similar to the findings of previous research (Apostolou et al., 2024), we found that in the Lithuanian cultural context, people frequently employed strategies aimed at improving their looks. In particular, the most likely to be used strategy was “Enhance looks,” while “Lose weight” was also a popular strategy. This finding indicates the importance that looks have in the mating domain, which is not surprising because looks summarize important fitness-related information, including genetic quality, health status, and age (Buss, 2016). Being able and competent is associated with the capacity to provide for one’s partner and children, so it is not surprising that “Show off abilities and talents” was the second most used strategy. Having good qualities may not matter much if partners are incompatible, so people attempt to demonstrate such compatibility to prospective mates, with “Demonstrate similarity” being the third most used strategy. People may also attempt to deceive prospective mates about their qualities using strategies such as “Show off and exaggerate wealth and abilities” and “Drastic appearance changes.” However, these strategies were reported to be the least frequently used.

Consistent with our original predictions and the results of previous research (Apostolou et al., 2024), women employed the “Enhance looks” and “Lose weight” strategies aimed at improving appearance much more frequently than men. The prediction that men would use strategies aimed at demonstrating wealth and social status was partially supported. In particular, for “Increase income and social status” and “Show off and exaggerate wealth and abilities,” men reported more frequent use than women, but the effect was not significant. Nevertheless, the *p*-value was close to the significance level, suggesting that the effects would be significant if a larger sample were used.

A significant effect of age was found for most strategies in our sample. The effect was negative, indicating that older participants reported less use of the observed strategies than younger participants. We would expect the opposite effect to be present; that is, older individuals would have been involved in more relationships and, thus, would be more likely to have used the observed strategies. The direction of the effect is most likely explained by the fact that the observed strategies refer to attracting mates, and older individuals are likely to have been in long-term relationships or out of the mating market for a prolonged period, so they tend to forget what strategies they have used. To properly understand the effect of age, future studies need to ask participants who are actively looking for mates what strategies they currently use and see if there are differences between age groups.

Research on mate preferences has found consistency across cultures in what people prefer in a prospective mate (Buss and Schmitt, 2019). Thus, there should also be some consistency in the strategies that people use to become appealing as mates by addressing these preferences. For instance, if across different cultural settings people value good looks in a prospective partner, we would expect that across different cultures people would attempt to enhance their looks to become more appealing as mates. Consistent with this argument, we found that the factor structure of the strategies for becoming appealing as a mate in the Lithuanian cultural context was very similar to the one identified by Apostolou et al. (2024) using a cross-cultural sample. Thus, although more research is required, the findings of previous studies and the findings of the current study support the conclusion that across post-industrial societies, people use similar strategies for becoming appealing as mates. In a pre-industrial context, mate preferences are likely to differ from those in the post-industrial context. For instance, people in the former ascribe more importance to the family background of prospective mates than people in the latter (Apostolou, 2014). This means that the strategies people use in the pre-industrial context may not completely overlap with those they use in the post-industrial one. For example, in pre-industrial societies, people may attempt to demonstrate that they come from a good family background, a strategy that is not present in the post-industrial context. Future studies need to examine the strategies people use to become more appealing as mates in the pre-industrial setting and compare them with the strategies they use in the post-industrial setting.

The strategies people employ to become a more desirable mate depend on the type of intimate partner they wish to attract. For instance, individuals ascribe more importance to the good looks of a casual mate than to those of a long-term mate (Buss, 2016). Thus, when people seek short-term mates, they are more likely to use the strategy of enhancing their looks than when they seek long-term mates. Accordingly, future studies need to investigate how the adoption of these strategies varies with the type of relationship an individual wishes to establish. People need to look attractive to prospective mates to attract them, but they also need to remain attractive to their current mates to keep them. It follows that people are likely to employ strategies for remaining appealing to their current mates. The strategies for appealing to existing mates would, to some degree, overlap with the strategies for appealing to prospective mates; for example, people may enhance their looks to remain appealing to their current mates. Nevertheless, the two sets of strategies are also likely to diverge; for instance, strategies such as demonstrating similarity may not be used in the context of a current relationship. Thus, future research needs to examine the strategies that people use to remain appealing to their current mates.

One limitation of the current research is that it was based on self-report data, which are subject to several biases, including participants giving inaccurate answers. For instance, they may be unwilling to admit that they would use strategies that involve deception, such as “Show off and exaggerate wealth and abilities.” Moreover, we employed a non-probability sample, so our findings may not readily generalize to the population. Additionally, we employed a previously developed instrument, which, although inclusive, may not capture acts of self-promotion that are exclusive to the current cultural context. Furthermore, factors other than sex and age may predict the observed strategies. For instance, individuals, especially men, with high socioeconomic status may demonstrate this capacity more than those who do not score high in this dimension. Future studies need to examine the effect of additional factors on the observed strategies.

People have a battery of strategies that they employ to become more appealing as mates. The current research found that enhancing one’s looks, demonstrating abilities and talents, as well as similarity with a prospective mate, were the most frequently used strategies in the Lithuanian cultural context. Yet, more research is necessary in other cultural settings if this complex phenomenon is to be better understood.

## Data Availability

The raw data supporting the conclusions of this article will be made available by the authors, without undue reservation.
